# Participation of Green Organs to Grain Filling in *Triticum turgidum* var *durum* Grown under Mediterranean Conditions

**DOI:** 10.3390/ijms19010056

**Published:** 2017-12-25

**Authors:** Othmane Merah, Philippe Evon, Philippe Monneveux

**Affiliations:** 1 Laboratoire de Chimie Agro-industrielle (LCA), Université de Toulouse, Institut National de la Recherche Agronomique (INRA), Institut National Polytechnique de Toulouse (INPT), 31030 Toulouse CEDEX 4, France; Philippe.Evon@ensiacet.fr; 2 Département Génie Biologique, Université Paul Sabatier, IUT A, 24 rue d’Embaquès, 32000 Auch, France; 3 International Potato Center/Centro Internacional de la Papa (CIP), Avenida La Molina 1895, La Molina, Lima 12, Peru; pmonneveux@yahoo.fr

**Keywords:** *Triticum turgidum* var. *durum*, carbon isotope discrimination, genetic variation, photosynthesis, re-mobilization, ear morphology

## Abstract

In wheat, flag leaf, stem, chaff and awns contribute to grain filling through photosynthesis and/or re-mobilization. Environmental and genetic effects on the relative contribution of each organ were examined by analyzing the consequences of sink-source manipulations (shading and excision) and by comparing carbon isotope discrimination (Δ) values in dry matter (at maturity) and sap (two weeks after anthesis) in six durum wheat genotypes grown in two contrasting seasons. The contribution of flag leaf, stem, chaff and awns to grain filling, estimated by sink-source manipulations, highly varied with the season. The contribution of ear photosynthesis and re-mobilization from the stem increased with post-anthesis water stress. They showed a large genetic variation that was, however, not clearly associated to morphological characteristics of ear and stem. Isotopic imprints of chaff on grain Δ were identified as a possible surrogate of the destructive and cumbersome sink-source manipulations to evaluate the contribution of carbon assimilated in ears or re-mobilized from stem. This might facilitate screening of genetic resources and allow the combining of favourable drought tolerance mechanisms in wheat.

## 1. Introduction

Several researchers have attempted to estimate the respective participation of current assimilation (resulting from photosynthesis productivity) and re-mobilization of pre-anthesis stored assimilates to winter cereal grain yield. Most studies were based on source–sink manipulation, sometimes complemented by the use of the heavy radioactive isotope ^14^C [1,2] or the stable isotope ^13^C [3,4]. Several works on grain filling have concentrated on the interaction between the source (provider of assimilates to the grain during filling) and the sink (the ability of kernel to incorporate the provided and available carbohydrates). Under favorable conditions, grain is mostly filled by current photoassimilates produced by green organs. When climatic conditions (mainly water deficit and high temperatures) are limiting, the contribution of photosynthesis to grain filling decreases and the remobilization of carbohydrates from senescent tissues rises. The wide variation observed in these estimations is likely to depend on the different protocols that have been used, but also relates to the effects of genotype and/or environment. There have been few attempts to quantify the impact of those factors on the relative contribution of different vegetative or reproductive organs to grain filling and final grain yield [5]. Sanchez-Bragado et al. [6] reported a higher contribution of ear to grain filling in landraces than in modern cultivars. In contrast, Maydup et al. [7] did not find any clear participation of awns to grain filling in bread wheat. Labelling by radioactive carbon isotope can permit us to determine part of the translocation and photosynthesis and determine the source of carbon for grain filling. However, this methodology is very difficult and tiresome to achieve. Stable isotope carbon (^13^C) is easier and more suitable for this approach. Carbon isotope discrimination (Δ) provides an integrated measure of photosynthetic gas exchange components and is therefore an integrated measure of transpiration efficiency during the entire period in which the sample tissue is growing. Moreover, values of Δ of different plant organs may reflect the variation of plant water status during the season, and the isotopic imprints of different organs on the final isotope composition of the grain could help advise their contribution to grain filling.

Under drought conditions, CO_2_ assimilation is more affected than the re-mobilization of carbon to the grain [8,9,10]. As a consequence, there is an increase in the contribution of stored dry matter to grain filling relative to current assimilation [11]. The contribution of the re-mobilization of carbon products stored in vegetative parts may vary between 5% and 10% under near optimal conditions [12] to 40% and 60% under water stress [13]. Merah and Monneveux [14] estimated this contribution to be 19.4–29.9%. There are two components involved in the contribution of stored assimilates to grain yield in wheat: i.e., the ability to store assimilates in stem and other plant parts, and the efficacy with which these stored reserves are re-mobilized and transported into the grain [11,15]. Both components depend on genetic factors [16,17].

The relative contribution of ear (spike and awns) photosynthesis to final grain weight varies between 10% and 76% according to environmental and genetic factors [11,18]. Ear acquires a major role in photosynthesis and yield under drought [19] and high temperature [20] conditions. These studies did not emphasize the environmental and genotype effects on the contribution of different organs to grain filling. Indeed, most of these works have been carried out on one genotype under controlled conditions. In the present article, these effects were estimated by analyzing the effects of source–sink manipulations on grain weight and using the stable isotope ^13^C as an indicator of current photosynthesis and re-mobilization [14,21,22]. Associations between the contribution and carbon isotope discrimination (Δ) values of the different organs and their morphological characteristics were examined under rainfed conditions on six field grown genotypes of durum wheat. The relationships between the contribution of Δ of the different organs to grain Δ (expressed as the difference between the two values) and the morphological characteristics of these organs were also analyzed.

## 2. Results

### 2.1. Variation of Morphological and Agronomic Traits

The main morphological characteristics of the six cultivars used in the study are given in Table 1. In the two seasons, Caravaca Colorado was the tallest cultivar and Casablanca 7580 and Brachoua/*Triticum dicoccoides*-SY20017//Haucan were the shortest. Grain yield (GY) and thousand kernels weight (TKW) were strongly correlated in both seasons. Shading and excision had highly significant effects on the GY, NGS and TKW of the six cultivars in both seasons (Table 2); shading effects, however, were higher than those of excision. Effects of shading and excision were more pronounced in Season 2 than in Season 1.

### 2.2. Effect of Shading and Excision Treatments on Grain Filling

**Figure 1:**
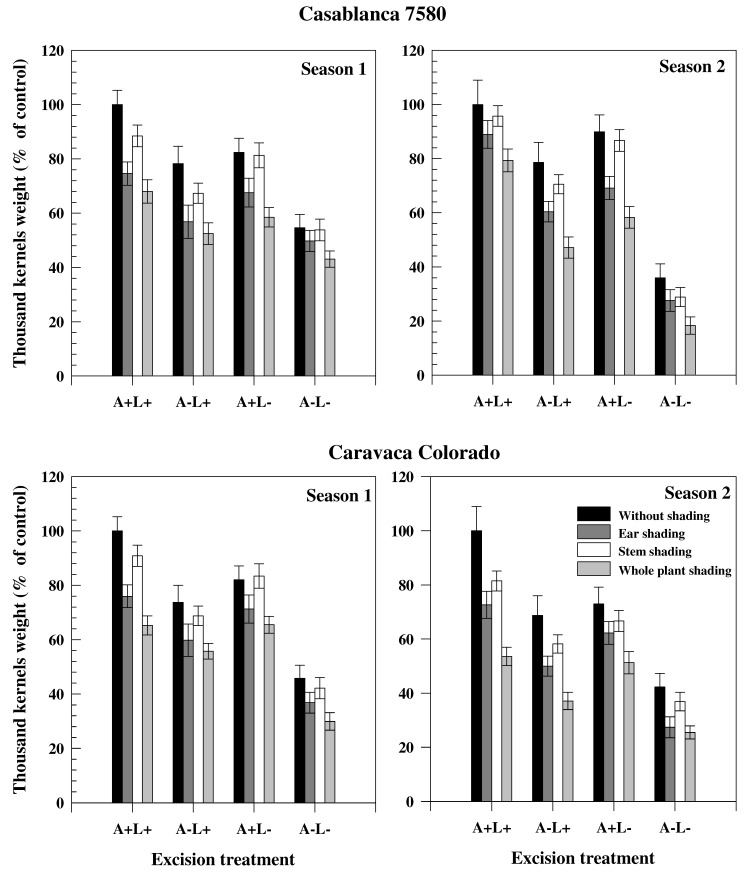
Thousand kernels weight (expressed in % of control plant –without shading and excision) of two durum wheat genotypes grown under rainfed conditions during two consecutive growth seasons. Excision treatments were performed by cutting leaves or awns or both. Shading was done by darkening of spikes (+awns if not excised), stem (+leaves if not excised) or whole plant.

The decrease of TKW (expressed in % of the control plants) in the different seasons and treatments is presented in Figure 1 for the cultivars Casablanca 7580 and Caravaca Colorado. In both genotypes, TKW was higher in Season 2 than in Season 1. Stem, ear and plant shading led to a significant reduction of TKW, independently of the season or cultivar. Ear shading, however, was associated with a higher decline in TKW than stem shading. The reduction in TKW induced by ear shading was 21.7% and 26.3% in Casablanca 7580 and Caravaca Colorado, respectively, in Season 1, and 21.4% and 31.3% in Season 2. Average TKW reduction across all treatments involving ear shading (treatments B, F, G and N) was similar in Casablanca 7580 and Caravaca Colorado in Season 1 (36.3% and 35.5%, respectively). Conversely, in Season 2, the average TKW for ear shading treatments was 35.8% lower in Casablanca 7580 and 46.5% lower in Caravaca Colorado compared to the control plants. Awn excision reduced TKW to the same extent in Casablanca 7580 (37.8%) and Caravaca Colorado (39.0%) in Season 1, but had a higher impact on Caravaca Colorado (46.9%) than Casablanca 7580 (38.5%) in Season 2. Flag leaf excision affected similarly the two cultivars. The contribution to grain filling (in %) of stem, flag leaf, spike and awns, estimated from different treatments, is presented in Table 3 for the two cultivars. The major contribution to grain filling came from stem and spike that participated oSn average (both through photosynthesis and re-mobilization) for more than 70% to final grain weight. Contribution of spike and stem photosynthesis showed a broad variation with values ranging from 9.5% to 41.6% and 2.3% to 57.8%, respectively, while flag leaf photosynthesis contributed less than 12.2%. The participation of ear (spike and awns) to grain filling was also high, but spike participated more than awns, particularly in photosynthesis. The participation of flag leaf (both through photosynthesis and re-mobilization) was lower than the participation of awns. The contribution of the spike was slightly increased in Season 2, compared to Season 1. The contribution of stem, although similar in both seasons in Caravaca Colorado, was much higher in Casablanca 7580 in Season 2 than in Season 1.

### 2.3. Effect of Treatments on Carbon Isotope Discrimination of Plant Parts

**Figure 2:**
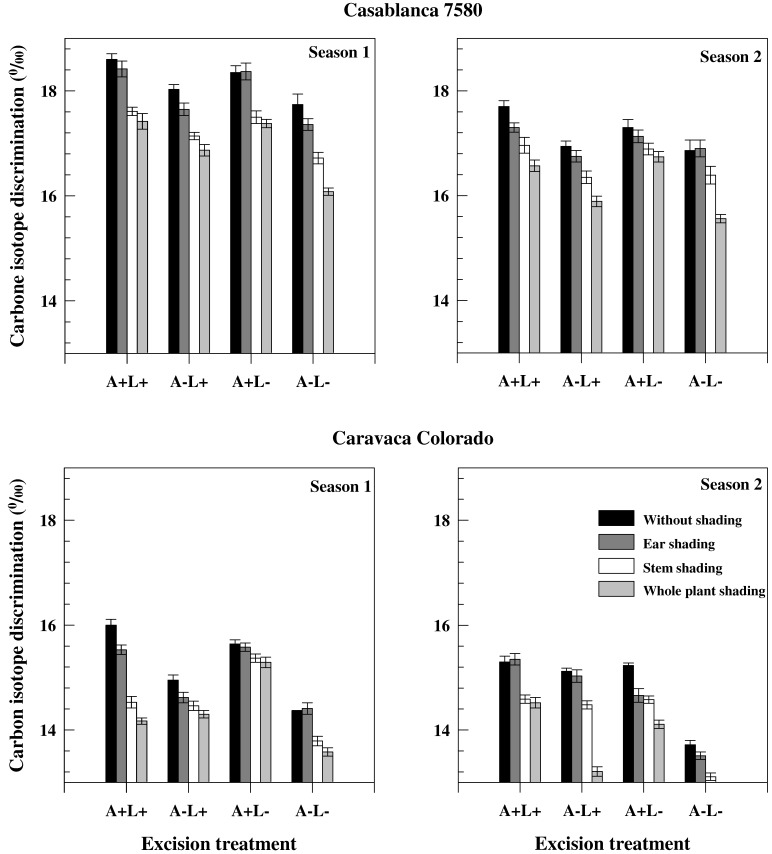
Grain carbon isotope discrimination values of the different treatments carried out on two durum wheat genotypes grown under field conditions during two consecutive growth seasons.

Grain carbon isotope discrimination (ΔG_m_) was, on average, higher in Season 1 than in Season 2 and in Casablanca 7580 than in Caravaca Colorado (Figure 2). It was, however, less affected by the different treatments than TKW (Figure 1 and Figure 2). Decrease of ΔG_m_ induced by stem shading was higher than the decrease caused by ear shading. Ear shading and awn excision (when not associated with flag leaf excision) were associated with significant differences in ΔG_m_ between seasons in Casablanca 7580, but not in Caravaca Colorado. Carbon isotope discrimination values were lower in sap than in dry matter (Table 4). Carbon isotope discrimination in sap (Δ_s_) was lower in chaff and awns than in leaves and in sap of chaff (ΔC_s_) and awns (ΔA_s_), both in Casablanca 7580 and Caravaca Colorado. ΔC_s_ also correlated with TKW and yield. ΔA_s_ correlated with GY in Casablanca 7580 but not in Caravaca Colorado.

### 2.4. Relationships between Traits

**Figure 3:**
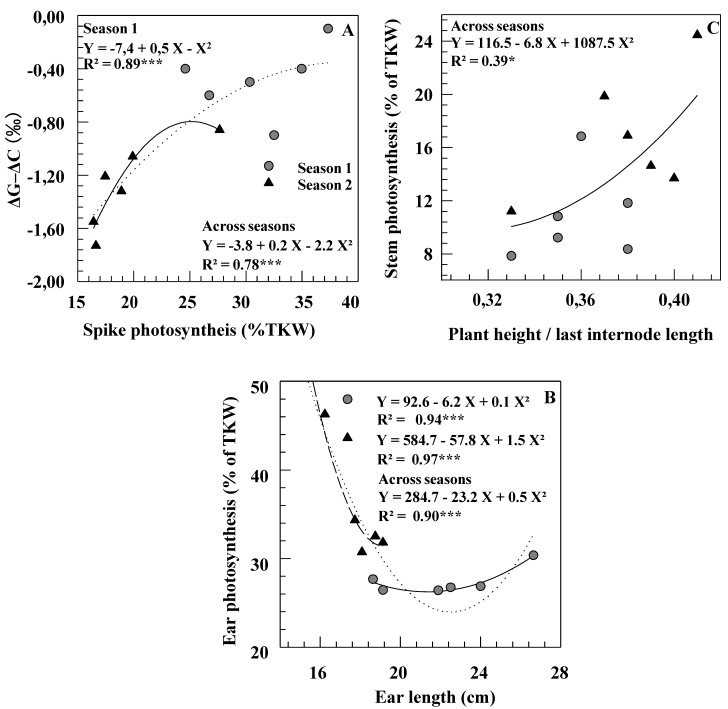
Relationships between photosynthesis (expressed in percent of thousand kernels weight) and ΔG–ΔC (**A**), morphological traits of ear (**B**) and of stem (**C**) measured on six durum wheat genotypes grown under rainfed conditions during two consecutive growth seasons.

A significant correlation across the six cultivars was noted in both seasons between grain carbon isotope discrimination (ΔG_m_) and yield of the control plants (r = 0.84 and r = 0.80, *p* < 0.01, respectively). In Season 1, a significant correlation was found between ΔG_m_–ΔC_m_ (i.e., the isotopic signatures of chaff on mature grain) and the contribution of spike photosynthesis to grain filling (Figure 3A). Within and across seasons, ear length and ear photosynthesis contribution were negatively associated (Figure 3B). In Season 2, the calculated contributions of different organs did not relate with TKW, suggesting that no organ had, under these conditions, a determining impact on grain filling. A positive correlation was noted between LIL/PH ratio and stem photosynthesis contribution across season and genotypes (Figure 3C).

## 3. Discussion

The differences in GY, TKW and ΔG_m_ between the two seasons were related to the amount of precipitation during the growing season and are in agreement with those reported for a wider set of genotypes tested concurrently [15,23]. RWC values observed in this study (Table 1) were lower than those observed by Merah [24] on a large collection of durum wheat genotypes under favourable conditions. This mirrored that these genotypes under these conditions have suffered a moderate water deficit at anthesis [23,24]. TKW and GY were lower in Season 1 as a consequence of the strong post-anthesis water stress experienced by the crop (Figure 1). Conversely, ΔG_m_ was higher in Season 1, probably because of the enhanced re-mobilization of C products having high Δ value, contributing to an increase of Δ in the grain (Figure 2 and Table 4). The correlation across genotypes between ΔG_m_ and yield (r = 0.84, *p* < 0.01) confirmed previous [24,25,26] and was stronger when rainfall distribution corresponded to typical post-anthesis water stress (i.e., Season 1), as has already noted by Monneveux et al. [25] in wheat and Tsialtas et al. [27] in dry bean. The close association between GY and dry matter Δ across treatments confirmed the potential interest of Δ as an indicator of yield effects of source–sink manipulations [3,21]. Carbon isotope discrimination in sap (Δ_s_) was lower in chaff and awns than in leaves in both cultivars, and significant correlations were found between Δ_s_ and dry matter Δ (r = 0.903, *p* < 0.05 in Casablanca 7580 and r = 0.962, *p* < 0.01 in Caravaca Colorado). ΔC_s_ was also correlated with TKW and GY. ΔA_s_ correlated with GY in Casablanca 7580 but not in Caravaca Colorado. All told, the data suggest that ΔC_s_ could also be used as an indicator of yield effects of source-sink manipulations.

The high decline in TKW induced by ear shading confirmed the high contribution of ear photosynthesis to grain filling (Figure 1). The contribution of spikes was slightly increased in Season 2, possibly because of the capacity of spike organs to better tolerate water stress than leaves. Spikelets can maintain higher water potential than leaves under water stress conditions and are more effective at osmotic adjustment [26]. An analysis of the role of ear parts in grain filling was attempted by Araus et al. [28] in triticale (× *Triticosecale* Wittmack) by comparing Δ values in grain and of water extracts of ear bracts, awns and flag leaves. Results indicated that a substantial portion of the photosynthetates in the grain came from ear parts. The lower Δ values in ear parts (Table 4) also suggested higher transpiration efficiency, probably related to some distinct xeromorphic features, such as thick epidermis and cuticule in the dorsal part of ear bracts [29,30]. 

Contribution of ear and awns to grain filling was greater in Casablanca 7580 than in Caravaca Colorado, particularly in the driest season (Table 3). The relationships observed here between ear photosynthesis and ear length within and across seasons (Figure 3B) indicate that, under some conditions (Season 2, short ears), the photosynthetic capacity of the ear is a function of its total area, including the awns [5,31]. Chhabra and Sethi [32] found a significant positive correlation between awn length and contribution to yield across eleven durum wheat genotypes. In the present study, however, Casablanca 7580 had shorter spikes and awns than Caravaca Colorado (Table 1) and the relative Δ values of ear organs did not clearly relate to spike morphology (Table 4). In contrast, Casablanca 7580 was found to have higher Δ (lower TE) in chaff, but lower Δ (higher TE) in awns than Caravaca Colorado (Table 4). Combined results indicate that genetic variation in yield contribution of the ear might depend not only on morphological features, but also on differences in ear transpiration efficiency [4,5].

Contribution from stem re-mobilization was higher in the driest season [1,2,8]. Thus, a higher amount of reserves stored in vegetative organs before anthesis and available for later translocation to the grain could buffer grain yield against environmental stresses [1,17,33]. The increase in the contribution of re-mobilization from the stem with drought conditions was more noticeable in the short statured cultivar Casablanca 7580 than in Caravaca Colorado. However, no relationship was found in the set of six genotypes between contribution of stem re-mobilization and plant height (Figure 3C). These results confirmed that tall cultivars were not more efficient than dwarf ones at utilizing their reserves to grain filling [22,34]. Moreover, some short genotypes have shown even better re-mobilization efficiency than tall ones [17,33]. This suggests, as postulated by Zhu et al. [11], that the relative importance of the contribution of stem reserves to final yield is related to more efficient re-mobilization of C-products (possibly related to sink strength) more than to morphological characteristics of the plant (such as stem height).

The estimated contribution of leaves to photosynthesis and re-mobilization showed variation with season, despite the strong reduction of leaf area in Season 2 (−15.2%) (Table 3). Chhabra and Sethi [32] reported that removal of flag leaf affected more adversely GY in dwarf genotypes than in taller ones. In the present work, leaf area was 37% and 41% greater in Caravaca Colorado than in Casablanca 7580 and the contribution of leaves to grain filling did not differ between the two cultivars (Table 1). Martinez et al. [35] reported that, under drought, leaves showed a great decrease of photosynthetic activity compared to ears allowing to a low participation of the leaves to grain filling.

## 4. Materials and Methods

### 4.1. Plant Material

The genotype effect on the contribution of different organs to grain filling was first analyzed on two durum wheat (*Triticum turgidum* var. *durum*) cultivars, Casablanca 7580 and Caravaca Colorado, having shown contrasted carbon isotope discrimination (Δ) values in previous studies [3,15]. Casablanca 7580 is a semi-dwarf advanced line, with short spikes and awns. Caravaca Colorado is a tall-statured landrace from Portugal. It has a very long spike and very large leaf area. These two cultivars have similar precocity. The genetic variation in the contribution of some organs to grain filling, as well as the possible association of contribution with morphological traits was then confirmed on a set of six genotypes, including the two precedent ones as well as Camadi-Abou, Cakmak, Brachoua/*Triticum dicoccoides*-SY20017//Haucan, and Blk2/4/134XS-69-186/368/1/5/Mrb9/6/Awalbit-3.

### 4.2. Experimental Conditions

**Figure 4:**
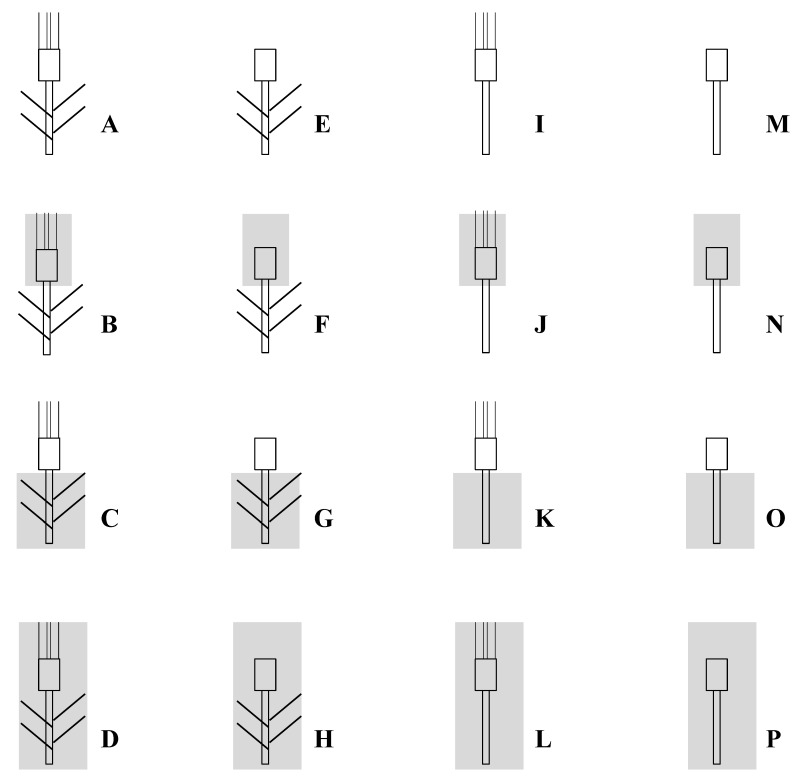
The different treatments based on excision and/or shading used to study sink-source relations on six durum wheat genotypes grown under field Mediterranean conditions.

The experiments were carried out in the ENSA-INRA Montpellier (South of France) experimental fields (48°46′ N, 4°29′ E, 45 m asl). The soil was a sandy-loam (organic matter content 2.1%, pH 7.8) with a depth of about 0.6 m. The six cultivars were grown under rainfed conditions during two successive growth seasons (1995/96 and 1996/97), hereafter referred to as Season 1 and Season 2; sowings occurred on 17 and 8 November, respectively. Full tillering stage was reached at early March. Anthesis occurred at the first week of May, and maturity was attained at the end of June. A randomized complete block design was used for the two trials, with four replicates per genotype. Seeds were sown in plots of 18 m^2^ (6 m long × 3 m wide; 25 cm spacing row and 3 cm inter-plant spacing). Within each plot, tree plants were used for each treatment as described in Figure 4. For example, treatment M (leaves and awns excised without shading) was repeated three times within each plot. Fertilizer was applied before sowing at 90 kg N ha^−1^, 90 kg P ha^−1^ and 30 kg K ha^−1^. The trials were top-dressed at the onset jointing and the beginning of heading with 70 and 20 kg N ha^−1^. Pests and diseases were controlled with chemicals.

Climatic conditions are presented in Table 5. The distribution of precipitation was quite different between the two seasons. Both seasons were characterized by a high level of precipitation during the period from sowing to tillering (62% and 74% of the precipitation in Seasons 1 and 2, respectively), which exceeded largely the evaporative demand (Table 5). When plants reached full tillering stage, the amount of precipitation decreased in Season 1. This decrease was more pronounced during the period from anthesis to maturity (i.e., grain formation and filling) comprising only a 7% of water received by plants during the season. In contrast, Season 2 was characterized by a low precipitation to evapotranspiration ratio (Table 5). This low ratio mirrored that the evaporative demand was 3.5 higher than precipitation. Moreover, average temperature during this growth stage was higher in Season 2 than in Season 1. In Season 2, plants received 18% of the precipitation during the final growth stage, an amount equilibrating the evaporative demand (Table 5). Thus, Season 1 was mainly characterized by well-watered conditions during pre-anthesis period and a strong water stress after anthesis. Conversely, moisture stress in Season 2 began before anthesis and intensified during the major part of grain filling.

### 4.3. Treatments and Measurements

At anthesis, plant height (PH), last internode length (LIL), ear (spike + awns) lengths were measured on the six cultivars, and the LIL to PH ratio (LIL/PH) was calculated. Flag leaf area was estimated on 10 plants per plot using a planimeter (LI-3000, Li-Cor, Lambda Instruments, Co., Lincoln, NE, USA). One week after anthesis, 16 different treatments consisting of excision or shading different organs, as performed by Merah [15] where applied (Figure 4). Two weeks after anthesis and in Season 2, two plants of each treatment were used to determine carbon isotope discrimination (Δ) in cell sap of chaff, awns, flag leaves (if not excised) and stems. Measurements of carbon isotope composition of samples were achieved with an elemental analyser (Carlo-Erba, Courtaboeuf, France) coupled to an isotope mass spectrometer (Micromass, Villeurbanne, France) operating in continuous flow mode allowing the determination of the isotopic ratio ^13^C/^12^C of the same samples as:

δ^13^C (‰) = [(R sample/R reference − 1) × 1000],

R being ^13^C/^12^C ratio.

The standard error was 0.1‰. Carbon isotope discrimination (Δ) was calculated using the following formula [36]:

Δ (‰) = [(δa − δp)/(1 + δp)] × 1000,

where δp is the carbon isotope composition (δ^13^C) of the samples and δa, the δ^13^C of the atmospheric CO_2_, −8‰.

Plants of each combination were harvested at maturity. Days to anthesis (number of days from sowing to anthesis), grain yield per plant (GY), number of grains per spike (NGS) and thousand kernel weight (TKW) were determined for each treatment, cultivar and season. By comparing the different treatments, we calculated the percent of contribution of stem, flag leaf, spike and awns photosynthesis and re-mobilization to TKW. These contributions were estimated from different combinations of two or three treatments, as presented in Table 6. The overall contribution of flag leaf and awns to grain yield was evaluated by excising awns or flag leaves or both. Decrease in yield produced by leaf excision may be due not only to the elimination of a photosynthetic function of this organ, but also to the disappearance of a potential intermediate reserve. The role of whole plant, ears and stem photosynthesis was examined by covering them with aluminium sheet. The sheet was holed to prevent accumulation of ethylene and water vapour.

For example:

photosynthesis of awns = [(C-D)-(G-H)]
C = photosynthesis of ear (spike and awns) + remobilization of stem and leaves
D = remobilization of whole plant (including ear remobilization)
C-D = photosynthesis of ear
G = photosynthesis of spike + remobilization of stem and leaves
H = remobilization of spike + leaves + stem
G-H = photosynthesis of spike
[(C-D)-(G-H)] = photosynthesis of awns.

Grain, stems, chaff, flag leaves and awns (if not excised) were collected separately. All samples, including sap samples, were oven-dried at 80 °C for 48 h and plant material was ground to fine powder for carbon isotope analysis. Carbon isotope discrimination values in dry matter (Δ_m_) of stem, flag leaf, chaff, awns and grain were hereafter referred to as ΔS_m_, ΔL_m_, ΔC_m_, ΔA_m_ and ΔG_m_, respectively. The variation of ΔG_m_ with season and treatments, and its relationship with TKW and GY, was analyzed in Casablanca 7580 and Caravaca Colorado. The relative influences of Δ_m_ values in stem, flag leaf, chaff, awns in determining ΔG_m_ value (or imprint of ΔS_m_, ΔL_m_, ΔC_m_ and ΔA_m_ on ΔG_m_) were estimated in the six cultivars as the differences between ΔG_m_ and ΔS_m_, ΔL_m_, ΔC_m_ and ΔA_m_, respectively [21], with low (negative) values reflecting stronger imprints. The across-cultivars relationship between the imprint of a given organ and its morphological characteristics was analyzed. Carbon isotope discrimination values in sap (Δ_s_) of stem, flag leaf, chaff and awns were referred to as ΔS_s_, ΔL_s_, ΔC_s_ and ΔA_s_, respectively. Detailed information on Δ measurements has been presented in Merah and Monneveux [14]. In Season 2 only, two plants per plot of each treatment were sampled to determine carbon isotope discrimination in cell sap. Chaff, awns, flag leaves (if not excised) and stems were sampled two weeks after anthesis, a stage which corresponds to the onset of the linear phase of grain filling. The cell sap was obtained by pressing the organ in a plastic syringe according to Hannachi et al. [21]. The sap was dried at 60 °C and measurements of Δ were made as for dry matter.

### 4.4. Statistical Analysis

Effects of cultivar and season were estimated for Casablanca 7580 and Caravaca Colorado using GLM procedure of SAS. Comparison for morphological, agronomic, precocity, TKW and ΔG_m_ between different combinations of control, shading and excision were performed using Tukey test at 5% probability level. Cultivars and environments were considered as fixed. The relationships between the contribution of a given organ, the imprint of its Δ_m_ value on ΔG_m_ and its morphological characteristics were analyzed across the six genotypes by single correlation within each year and across years using CORR procedure of SAS.

## 5. Conclusions

The results of the present study showed that the contributions of different organs, as well as the respective role of photosynthesis and re-mobilization, are highly influenced by environmental and genetic factors. They confirmed that, under typical post-anthesis water stress, ear photosynthesis plays a major role in grain filling. A rapid evaluation of this trait can be provided by Δ values. Because of the cumbersome character of source–sink manipulations, the present study has been carried out on a limited number of cultivars differing in many morpho-physiological traits. The association between the isotopic imprint of chaff Δ on ΔG_m_ should be consequently verified on a greater number of genotypes and validated by divergent selection.

## Figures and Tables

**Table 1:** The main morphological, physiological, agronomic characteristics and precocity of the six genotypes studied (LSD: least significant difference).

Genotype	Origin	Type	PH (cm)	LIL (cm)	LIL/PH	EL (cm)	LA (cm^2^)	DH (Days)	TKW (g)	NGS	GY (g.plant^-1^)	RWC (%)
*Season 1*												
Casablanca 7580	Portugal	L	92.00 ^d^	35.00 ^c^	0.38 ^a^	18.63 ^c^	25.01 ^b^	134	47.75 ^b^	44.00 ^b^	13.66 ^a^	86.12 ^a^
Brachoua/*Triticum dicoccoides*-SY20017//Haucan	CIM/ICAR	AL	93.50 ^d^	33.08 ^c^	0.35 ^b^	19.13 ^c^	23.32 ^b^	133	46.24 ^b^	38.75 ^c^	7.61 ^d^	82.99 ^b^
Blk2/4/134XS-69-186/368/1/5/Mrb9/6/Awalbit-3	CIM/ICAR	AL	132.00 ^b^	45.83 ^a^	0.35 ^b^	21.88 ^b^	21.57 ^b^	133	48.40 ^b^	49.54 ^a^	12.69 ^b^	84.78 ^b^
Camadi-Abou	Portugal	L	126.05 ^b^	41.57 ^b^	0.33 ^c^	22.50 ^b^	21.67 ^b^	138	46.87 ^b^	38.51 ^c^	8.44 ^d^	87.91 ^a^
Cakmak	Turkey	L	117.25 ^c^	44.18 ^b^	0.38 ^a^	24.05 ^b^	25.21 ^b^	137	46.41 ^b^	45.06 ^b^	7.82 ^d^	88.45 ^a^
Caravaca Colorado	Portugal	L	162.75 ^a^	58.59 ^a^	0.36 ^b^	26.63 ^a^	34.30 ^a^	136	55.38 ^a^	45.75 ^b^	9.70 ^c^	85.43 ^a,b^
LSD			6.02	3.14	0.02	2.05	4.01	ns	3.11	3.48	0.72	2.9
*Season 2*												
Casablanca 7580	Portugal	L	86.17 ^c^	35.28 ^b^	0.41 ^a^	16.23 ^c^	17.39 ^b,c^	136	44.43 ^b^	43.72 ^a,b^	13.06 ^a^	84.72 ^b^
Brachoua/*Triticum dicoccoides*-SY20017//Haucan	CIM/ICAR	AL	83.17 ^c^	31.48 ^c^	0.38 ^a,b^	17.73 ^b^	21.11 ^b^	136	41.89 ^b,c^	35.10 ^c^	9.15 ^b^	89.43 ^a^
Blk2/4/134XS-69-186/368/1/5/Mrb9/6/Awalbit-3	CIM/ICAR	AL	95.40 ^b^	37.45 ^b^	0.39 ^a^	18.75 ^a,b^	20.74 ^b^	136	47.10 ^b^	46.87 ^a^	12.36 ^a^	87.71 ^a,b^
Camadi-Abou	Portugal	L	90.40 ^b,c^	33.62 ^c^	0.37 ^b^	19.13 ^a^	16.65 ^c^	140	39.44 ^c^	39.10 ^b^	9.66 ^b^	90.59 ^a^
Cakmak	Turkey	L	98.80 ^b^	39.89 ^a^	0.40 ^a^	15.28 ^c^	27.70 ^a^	140	40.78 ^c^	43.85 ^a,b^	9.76 ^b^	92.78 ^a^
Caravaca Colorado	Portugal	L	123.07 ^a^	41.15 ^a^	0.33 ^c^	18.08 ^b^	24.57 ^a^	137	54.90 ^a^	42.42 ^b^	12.97 ^a^	87.89 ^a,b^
LSD			5.98	3.11	0.02	1.04	3.38	ns	4.01	3.41	0.69	3.45

CIM/ICAR = CIMMYT/ICARDA program, L = Landrace, AL = Advanced Line, PH = plant height, LIL = last internode length, LIL:PH = last internode length to plant height ratio, EL = Ear length (awns + spike), LA = leaf area, DH = Days from sowing to heading, TKW = Thousand kernels weight, NGS = Number of grains per spike, GY = grain yield/plant, RWC = Relative water content. Within each trial, means followed by a different letter are significantly different by Duncan’s test at *p* < 0.05; ns: not significant.

**Table 2:** Effect of shading and excision treatments on grain yield (GY), number of grain per spike (NGS) and thousand kernels weight (TKW) across the six cultivars, in Seasons 1 and 2.

Treatment	GY (g/Plant)		NGS		TKW (g)	
	Effect	Mean	Effect	Mean	Effect	Mean
*Across seasons*						
Control		10.6		42.7		46.6
Shading	1065.69 ***	5.5	14.41 ***	37.5	1469.89 ***	29.0
Excision	333.51 ***	5.8	7.08 ***	37.8	436.97 ***	30.2
*Season 1*						
Control		10.0		43.6		48.5
Shading	645.54 ***	5.2	8.44 ***	35.3	589.21 ***	30.7
Excision	202.02 ***	5.3	4.15 **	36.1	175.16 ***	31.1
*Season 2*						
Control		11.2		41.8		44.8
Shading	596.32 ***	5.8	5.03 **	39.8	994.58 ***	27.3
Excision	278.65 ***	6.3	2.72 *	39.3	231.89 ***	29.4

*, **, *** significant at 0.05, 0.01 and 0.001 of probability levels, respectively. *F*- and mean values are presented for the traits measured in the two seasons and their over year combination.

**Table 3:** Estimation of the relative contribution (expressed in %) of stem, leaf, spike, and awn photosynthesis and re-mobilisation to thousand kernel weight in cultivars Casablanca 7580 and Caravaca Colorado.

Genotype	Season	Stem	Leaves	Spike	Awns
*Casablanca 7580*					
Photosynthesis	Season 1	7.1–13.5	0–10.4	15.4–17.8	9.9–12.1
	Season 2	9.0–57.8	0–1.1	28.5–41.6	0–17.9
Re-mobilization	Season 1	9.3–13.6	0.8–10.1	15.5–17.8	4.0–21.4
	Season 2	28.8–41.6	1–10.9	28.6–32.2	8.4–23.4
*Caravaca Colorado*					
Photosynthesis	Season 1	7.5–26.6	4.6–10.5	9.5–35.6	1.8–5.7
	Season 2	2.3–21.3	8.1–12.2	16.5–34.9	0.4
Re-mobilisation	Season 1	16.8–17.7	1.9–6.9	9.6–16.2	9.0–14.8
	Season 2	11.6–21.2	5.1–11.0	16.5–22.7	6.8–21.1

**Table 4:** Carbon isotope discrimination of dry matter and cell sap determined on different plant of the cultivars Casablanca 7580 and Caravaca Colorado in Season 2. For each genotype, means followed by a different letter are significantly different by Duncan’s multiple range test at *p* < 0.05. LSD: least significant difference.

Plant Organ	Casablanca 7580		Caravaca Colorado	
	Dry Matter	Cell Sap	Dry Matter	Cell Sap
Stem	19.0 a	18.5 b	17.1 a	16.8 b
Flag leaf	19.4 a	18.8 b	17.3 a	16.7 b
Chaff	17.6 a	17.4 a	15.9 a	15.7 a
Awns	17.8 a	17.6 a	16.8 a	16.0 b
Grain	17.7 a	16.6 b	15.3 a	14.7 b

**Table 5:** Mean values of temperature (T), cumulative values of rainfall (R) and of evapotranspiration (ET) as well as their ratio (R/ET) at different growth stages of the six wheat genotypes during the two growth seasons.

	Season 1				Season 2			
Period of Development	T (°C)	R (mm)	ET (mm)	R/ET	T (°C)	R (mm)	ET (mm)	R/ET
Sowing—Tilling	9.5	578	39	14.8	9.7	548	30	18.3
Tilling—Anthesis	13.8	287	156	1.8	15.3	62	218	0.3
Anthesis—Maturity	19.7	68	222	0.3	19.1	134	158	0.9
Plant cycle	12.7	933	417	2.2	13.1	744	406	1.8

**Table 6:** Relative contribution of photosynthesis and re-mobilization of the different organs of durum wheat to the grain filling. Letters in column at right are the treatments (see Figure 4 for detail).

Contribution	Organ	Estimation
Photosynthesis	Flag leaf	[(A-C)-(I-K)]; [(E-G)-(M-O)]
	Ear	(C-D); (K-L); [(A-J)-(A-I)]; [(A-L)-(A-K)]
	Spike	(G-H); (O-P); [(A-F)-(A-E)]; [(A-H)-(A-G)]; [(A-N)-(A-M)]; [(A-P)-(A-O)]
	Awns	[(C-D)-(G-H)]; [(K-L)-(O-P)]; [(K-O)-(L-P)]; [(A-D)-(A-C)]
	Stem	(I-K); (M-O); [(A-K)-(A-I)]; [(A-L)-(A-J)]; [(A-O)-(A-M)]; [(A-P)-(A-N)]
Remobilization	Flag leaf	(C-K); (G-O); (H-P); (D-L); [(B-D)-(J-L)]; [(F-H)-(N-P)]
	Ear	(A-B); (I-J)
	Spike	(E-F); (M-N)
	Awns	(B-F); (D-H); (L-P); [(B-N)-(B-J)]; [(A-B)-(E-F)]; [(I-J)-(M-N)]
	Stem	(J-K); (N-P)

## Abbreviations

ΔS_m_	Carbon isotope discrimination in dry matter of stem
ΔL_m_	Carbon isotope discrimination in dry matter of flag leaf
ΔC_m_	Carbon isotope discrimination in dry matter of chaff
ΔA_m_	Carbon isotope discrimination in dry matter of awns
ΔG_m_	Carbon isotope discrimination in dry matter of grain
ΔS_s_	carbon isotope discrimination in sap of stem
ΔL_s_	carbon isotope discrimination in sap of flag leaf
ΔC_s_	carbon isotope discrimination in sap of chaff
ΔA_s_	carbon isotope discrimination in sap of awns
PH	Plant height
LIL	Last internode length
GY	Grain yield
NGS	Number of grains per spike
TKW	thousand kernels weight
